# Development of a Reliable, Valid Procedural Checklist for Assessment of Emergency Medicine Resident Performance of Emergency Cricothyrotomy

**DOI:** 10.5811/westjem.20365

**Published:** 2025-01-30

**Authors:** Dana E. Loke, Andrew M. Rogers, Morgan L. McCarthy, Maren K. Leibowitz, Elizabeth T. Stulpin, David H. Salzman

**Affiliations:** * University of Wisconsin School of Medicine and Public Health, BerbeeWalsh Department of Emergency Medicine, Madison, Wisconsin; † NorthShore University Health System, Division of Emergency Medicine, Evanston, Illinois; ‡ Northwestern University, Feinberg School of Medicine, Department of Emergency Medicine, Chicago, Illinois; § St Luke’s Hospital, Department of Emergency Medicine, New Bedford, Massachusetts; ¶ Icahn School of Medicine at Mount Sinai, Institute of Critical Care Medicine, New York, New York; # Emory University Hospital, Department of Emergency Medicine, Atlanta, Georgia; ∥ Northwestern University, Feinberg School of Medicine, Department of Medical Education, Chicago, Illinois

## Abstract

**Introduction:**

Emergency cricothyrotomy is a rare but potentially life-saving procedure performed by emergency physicians. A comprehensive, dichotomous procedural checklist for emergency cricothyrotomy for emergency medicine (EM) resident education does not exist.

**Objectives:**

We aimed to develop a checklist containing the critical steps for performing an open emergency cricothyrotomy, to assess performance of EM residents performing an open emergency cricothyrotomy using the checklist on a simulator, and to evaluate the reliability and validity of the checklist for performing the procedure.

**Curricular Design:**

We developed a preliminary checklist based on literature review and sent it to experts in EM and trauma surgery. A modified Delphi approach was used to revise the checklist and reach consensus on a final version of the checklist. To assess usability of the checklist, we assessed EM residents using a cricothyrotomy task trainer. Scores were determined by the number of correctly performed items. We calculated inter-rater reliability using the Cohen kappa coefficient. Validity was assessed using the Welch *t*-test to compare the performance of residents who had and had not performed an open emergency cricothyrotomy, and we used analysis of variance to compare performance of postgraduate year (PGY) cohorts.

**Impact/Effectiveness:**

The final 27-item checklist was developed after three rounds of revisions. Inter-rater reliability was strong overall (κ = 0.812) with individual checklist items ranging from slight to nearly perfect agreement. A total of 56 residents participated, with an average score of 14.3 (52.9%). Performance varied significantly among PGY groups (*P* < 0.001). Residents who had performed an emergency cricothyrotomy previously performed significantly better than those who had not *(P* = 0.005). The developed checklist, which can be used in procedural training for open emergency cricothyrotomy, suggests that improved training approaches to teaching and assessing emergency cricothyrotomy are needed given the overall poor performance of this cohort.

## BACKGROUND

Emergency cricothyrotomy is a rare but potentially life-saving procedure that emergency physicians (EP) must be able to competently perform. It is performed when the EP is unable to oxygenate and ventilate a patient after rapid sequence intubation is initiated and, therefore, must pursue cricothyrotomy in a time-sensitive manner. Thus, it is essential for EPs to be able to perform the procedure correctly. Furthermore, the Accreditation Council for Graduate Medical Education includes cricothyrotomy as a “key procedure” for which residents “must demonstrate competence.”^1^ However, there are few opportunities to learn this procedure in the clinical environment, with one study demonstrating that only 22% of graduating emergency medicine (EM) residents had the opportunity to perform cricothyrotomy on a living patient.^2^ Another study indicated that even experienced EPs felt that they lacked training in performing cricothyrotomy and that this procedural inexperience could directly affect the survival of a patient and lead to high emotional pressure.^3^ Lastly, the critically important nature of the procedure makes learning on shift a patient safety issue.

The combination of competency-based approaches using checklist-based assessments and the simulation environment has demonstrated a long track record of improving resident performance on specific procedural skills.^4^ ^–^ ^8^ While various instructional videos and checklists meant for different specialties are available, a standardized, reliable, valid, comprehensive, and dichotomous procedural checklist for assessment of performing emergency cricothyrotomy for EM resident education is lacking.^9^ ^–^ ^11^ Historically, the study site program’s method for teaching the open emergency cricothyrotomy occurred during the annual “rare procedures” simulation lab. These sessions involved non-standardized practice with a task trainer or sheep larynx that did not follow a competency-based training model.

## OBJECTIVES

Recognizing this unmet need in EM procedural training for our learners, we set several objectives in this study. The primary objective was to develop a checklist containing the critical steps for performing an open emergency cricothyrotomy based on input from a multidisciplinary team of experts. The second objective was to evaluate the reliability and validity of the checklist for performing open emergency cricothyrotomy. Finally, the third objective was to use the checklist to assess a group of EM residents on their ability to perform the procedure on a simulator and compare performance by training year.

## CURRICULAR DESIGN

### Checklist Development

We performed a literature review in MEDLINE and the MedEd Portal to assess published literature for emergency cricothyrotomy procedure checklists and curriculums. Key phrases for literature searches included “emergency cricothyrotomy curriculum,” “emergency cricothyrotomy checklist,” “emergency cricothyrotomy procedure,” “emergency cricothyrotomy simulation,” “emergency cricothyrotomy resident,” “emergency cricothyrotomy residency,” “emergency cricothyrotomy education,” and variations and combinations of the key words/phrases. Searches included all articles published until the search date of November 1, 2020. An EM procedural skills textbook and a surgical technique textbook were reviewed as well.^12^ ^,^ ^13^ We also evaluated relevant articles from the bibliographies of the textbooks and included studies for inclusion.

We used the Stufflebeam framework for checklist development after the literature review was completed.^14^ A preliminary dichotomous (“done” vs “incorrect/not done”) checklist was developed based on this literature review. The initial checklist was sent to a panel of 13 experts comprised of emergency physicians and trauma surgeons of varying practice type (academic, community, military), geographic practice location (within the United States), and gender. Practice type included 10 academic, two community, and one military hospital; practice location included five internal and eight external; and breakdown by sex was five female and eight male. Experts were blinded to each other’s identities and comments. We informed the expert panel of the curriculum’s intended audience of EM residents with anticipated use for a competency-based curriculum. We used a modified Delphi approach to serially refine the checklist and reach consensus on a final checklist.^15^ ^,^ ^16^ We then pilot-tested the checklist to ensure the items, wording, and formatting were ideally operationalized. Finally, the expert panel reviewed it for final approval.

### Study Population

The study was performed at a single urban academic center with a four-year EM residency training program. Four residents were excluded from the study due to their participation in the checklist design and assessment process. All other EM residents were included in the education as part of the annual simulation curriculum; however, participation in the study was voluntary. The study was reviewed by the institutional review board (IRB) at Northwestern University, Feinberg School of Medicine and determined to be exempt. Written informed consent was obtained from participants using a consent form approved by the IRB.

### Assessment

Assessments occurred in the simulation center using a simulation manikin (TraumaMan, Simlab, Seattle, WA) from August 31–September 28, 2021. Performance assessments were documented using an electronic version of the checklist in Qualtrics (Qualtrics, Seattle, WA), including a dichotomous “Yes” or “No” for completion of each step. One in-person rater (DL) was situated adjacent to the simulation manikin with the ability to move about the simulation room to ensure ideal visualization. Audiovisual recording of the assessment included one camera overhead providing a direct overhead view and a second camera situated to provide a view from the side. Each participant assessment was recorded from start to completion of the checklist. The dual video feeds with audio were saved as a single side-by-side video recording. These recorded videos were reviewed by a second rater at a later time. We used an online random number picker (https://www.random.org/lists/) to select 30% of the participants for scoring by the second rater.^17^ The second rater (AR) scored the randomly selected sample of video recordings using the same electronic assessment instrument in Qualtrics.

### Data Analysis

The checklist was analyzed for inter-rater reliability and validity among a cohort of EM residents ranging from PGY1-4. Inter-rater reliability was calculated overall and for each checklist step using the Cohen kappa coefficient. We determined validity using the Welch *t*-test to compare the performance of participants who had and had not performed an emergency cricothyrotomy in clinical practice or simulation and also between consecutive PGY groups. Analysis of variance was used to compare performance among PGY cohorts.

## IMPACT/EFFECTIVENESS

### Results

The literature search produced a total of 394 articles. After review, 13 articles were deemed suitable to inform checklist development. An additional two articles were identified and included upon reviewing references of the included articles and the two textbooks. We developed a preliminary 33-item dichotomous checklist based on this literature review. Consensus was achieved after three rounds of revisions, resulting in the fourth version of the checklist being the final version. We then tested the final 27-item checklist among ourselves for usability. Only minor wording and formatting changes were made to ensure ideal operationalization of the checklist. The final checklist was approved by the expert panel after usability testing, and no additional revisions were suggested.

The table includes percentage correct of checklist items, inter-rater agreement, and Cohen kappa coefficients for each checklist item. Overall, inter-rater reliability was strong (κ = 0.812) with individual checklist items ranging from fair to nearly perfect agreement, with one item having slight agreement. A total of 56 residents participated, including 15 PGY-1, 14 PGY-2, 13 PGY-3, and 14 PGY-4 residents. While only one resident had performed an emergency cricothyrotomy on a live patient, 69.6% had previously performed an emergency cricothyrotomy in simulation. The average checklist score for the overall resident cohort was 14.3 (52.9%). Emergency medicine resident checklist performance varied by PGY class (Figure). Performance varied significantly amongst PGY groups (*P* < 0.001). The PGY-4s performed best with an average score of 16.7 (61.9%) of checklist items completed correctly. They performed better than PGY-3s, but not significantly (61.9% vs 59.5%, *P* = 0.21). The PGY-3s performed significantly better than PGY-2s (59.5% vs 48.9%, *P* = 0.01). The PGY-2 performance was better but not significantly different compared to PGY-1 performance (48.9% vs. 42.7%, *P* = 0.13). The residents who had previously performed an emergency cricothyrotomy on a live patient or in simulation performed significantly better than those who had not (56.8% vs. 44.2%, *P* = 0.005).

**Table.** Percent correct, inter-rater agreement, and reliability for individual checklist-item scoring.

Checklist item		Percent correct	Rater agreement	Kappa coefficient
1.	Gathers sterile supplies	48.2%	64.7%	0.370
2.	Gathers primary cricothyrotomy procedure supplies	66.1%	100%	1.000
3.	Gathers secondary/supplemental cricothyrotomy procedure supplies	82.1%	94.1%	0.821
4.	Gathers supplemental intubation supplies	0%	100%	n/a^*^
5.	Washes hands	17.9%	94.1%	0.638
6.	Sterilizes the neck	87.5%	94.1%	0.767
7.	Dons personal protective equipment	67.9%	100%	1.000
8.	Proceduralist positions on the patient’s right side	89.3%	88.2%	0.605
9.	Identifies cricothyroid membrane (CTM)	482.%	52.9%	0.171
10.	Uses thumb and middle finger of non-dominant hand to stabilize airway	33.9%	88.2%	0.721
11.	Confirms incision site with palpation by index finger on the CTM using non-dominant hand while maintaining stabilization using thumb and middle finger of non-dominant hand	28.6%	88.2%	0.595
12.	Uses scalpel to make vertical skin incision ∼2–4 cm in length over the CTM using dominant hand	57.1%	64.7%	0.320
13.	Dissects down to CTM	87.5%	88.2%	0.433
14.	Re-identifies CTM by palpation or visualization	76.8%	100%	1.000
15.	Makes ∼1–2 cm (width of scalpel blade) horizontal incision through CTM with dominant hand and maintains scalpel blade in trachea	51.8%	76.5%	0.514
16.	Maintains patency of tract	12.5%	94.1%	n/a^*^
17.	Removes scalpel, only after tracheal hook, Trousseau dilator, bougie, or secondary scalpel handle is in place, maintaining patency of CTM	12.5%	94.1%	n/a^*^
18.	Proceduralist dilates CTM	3.6%	100%	1.000
19.	Inserts endotracheal tube or trach	91.1%	100%	1.000
20.	Inserts endotracheal tube or trach to correct depth	21.4%	88.2%	0.452
21.	Inflates the cuff with a 10-cc syringe	78.6%	88.9%	0.766
22.	Connects bag-valve-mask to endotracheal tube/trach and begins assisted ventilation	92.9%	94.1%	0.638
23.	Uses capnography to confirm tube location	89.3%	94.1%	0.638
24.	Listens for bilateral breath sounds	66.1%	94.1%	0.881
25.	Secures endotracheal tube/trach	64.3%	100%	1.000
26.	Orders chest radiograph	46.4%	100%	1.000
27.	Documents procedure	8.9%	100%	1.000

* Unable to calculate kappa coefficient due to one or both raters giving the same score to all scored participants.

**Figure.**
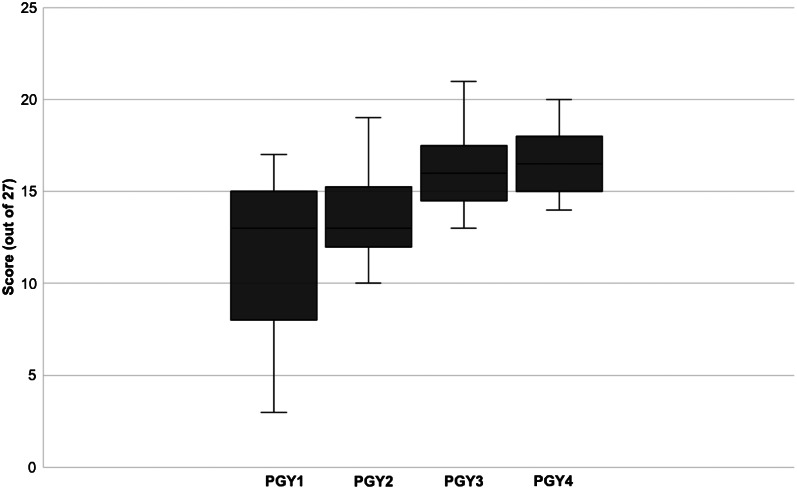
Emergency cricothyrotomy checklist performance by emergency medicine resident postgraduate year. Box limits represent the 25th and 75th percentiles with the median checklist score represented by the bar.*PGY*, postgraduate year.

### Discussion

Although we identified procedural narratives and checklists with varying degrees of specificity for our learner group at the time of our literature review, our search demonstrated a lack of a standardized, validated, reliable, and dichotomous procedural checklist for emergency cricothyrotomy for EM residents. This checklist adds to more recently published articles targeting attendings, students, and “novice” learners. This newly developed procedural checklist for emergency cricothyrotomy addresses this unmet need for EM resident procedural training.

The expert panel provided critical insight during the checklist development. Our initial checklist focused on the classic “hook and dilator,” scalpel-based approach to emergency cricothyrotomy. However, we ultimately revised the checklist based on expert feedback to include the additional accepted approaches of “scalpel only” and “bougie-assisted” emergency cricothyrotomy. The inclusion of all three accepted approaches allowed for a more versatile checklist that is more generalizable to all resource settings and better reflects the variable real-world environment and urgency of the procedure. The inclusion of multiple techniques also suggests generalizability to other clinical environments, such as surgery and otolaryngology; however, this was not the intended audience at the time the checklist was developed. While there are several potential options for performing an emergency cricothyrotomy, including a needle/wire Seldinger technique, this checklist reflects the development with the primary construct of using a scalpel-based approach.

This study’s strong overall inter-rater reliability using this checklist and one in-person rater and one remote-video rater reinforces previous studies using a similar technique.^18^ ^,^ ^19^ Additionally, inter-rater reliability using this method was strong overall, which is consistent with prior checklist development studies with similar methods.^18^ ^,^ ^19^ Most individual items had moderate to near-perfect inter-rater reliability, overall demonstrating reliability of the checklist.^20^ The items with the lowest kappa scores included “gathers sterile supplies” (item 1), “identifies cricothyroid membrane” (item 9), and “uses scalpel to make vertical skin incision ∼2–4 cm in length over the cricothyroid membrane using dominant hand” (item 12). We suspect that this likely reflects the remote nature of the second rater, as mishearing a request for a single piece of equipment or inability to accurately visualize the membrane or exact length of incision on a recorded video would lead raters to score differently. This could have been improved with greater verbalization of all steps by the learner and primary rater or having a second in-person rater when able.

The residents who had performed an emergency cricothyrotomy previously performed significantly better than those who had not, demonstrating criterion validity for this checklist as there was correlation with this group’s prior experience. Several studies with similar methods have also demonstrated congruent findings on checklist validity.^18^ ^,^ ^19^ While not significant, more senior PGY residents performed better as well. This may have been due to increased clinical exposure with seeing an emergent cricothyrotomy performed or improved procedural experience with practice in the simulation environment. However, despite these potential exposures and previous experiences, this cohort only correctly completed just over half of the checklist items.

Additionally, certain items had particularly low completion rate, including “Gathers supplemental intubation supplies” (item 4) (0%); “Proceduralist dilates cricothyroid membrane” (item 18) (3.6%); and “Documents procedure” (item 27) (8.9%). While some of these completion rates may be attributable to the simulation environment, it is important to highlight that merely planning for an intubation would not necessarily ensure that all equipment necessary for a cricothyrotomy was also available. The overall performance of this resident group, with residents only completing roughly 50% of the checklist items, suggests that the current, non-standardized technique for teaching emergency cricothyrotomy in this cohort is lacking and that a competency-based approach using a well-developed procedural checklist may improve performance.

## LIMITATIONS

This study has several limitations. First, the single-site nature of the study may not reflect resident performance at other institutions. Studying the checklist’s use at other residency sites would help to understand its generalizability to other environments with different approaches to teaching open cricothyrotomy. Second, while we recruited an expert panel including EM and trauma surgery representatives with diversity in practice type, practice location, and gender, most of the experts practiced in an academic environment. Despite this, the steps to performing the procedure should not vary by practice environment and, therefore, we do not believe that this limits validity or generalizability of the checklist. Expert panel review including additional community and hybrid experts would help test this hypothesis.

Third, the checklist and testing were performed using a bloodless simulation task trainer, which may not ideally represent an actual patient encounter. However, the infrequent nature of the procedure, as evidenced by only one resident having performed an emergency cricothyrotomy during their training, necessitates a non-clinical environment training simulation. While emergency cricothyrotomy simulation experience has been documented using sheep larynx and 3D-printed models, our study was not performed using these models and instead used a commercially available training device. Therefore, we do not know the influence of different simulation methods on the study and checklist performance, and this remains an area for future study.

## CONCLUSION

We designed a reliable, valid, dichotomous procedural checklist to assess EM residents’ ability to perform emergency cricothyrotomy. The overall performance of the residents tested in this study suggests that the current method of teaching emergency cricothyrotomy for this group is insufficient. Given the need to develop procedural competency for this rare but potentially life-saving procedure, a curriculum such as simulation-based mastery learning should be developed to ensure mastery of this procedure for EM residents. The checklist developed in this study could serve as a foundation for such a curriculum.
